# Elevated Levels of Plasma IgA Autoantibodies against Oxidized LDL Found in Proliferative Diabetic Retinopathy but Not in Nonproliferative Retinopathy

**DOI:** 10.1155/2016/2614153

**Published:** 2016-12-20

**Authors:** Satu Vavuli, Tuire Salonurmi, Sirpa Loukovaara, Antti E. Nissinen, Markku J. Savolainen, M. Johanna Liinamaa

**Affiliations:** ^1^PEDEGO Research Unit, Department of Ophthalmology, Medical Research Center (MRC Oulu), Oulu University Hospital and University of Oulu, Oulu, Finland; ^2^Research Unit of Internal Medicine, Medical Research Center Oulu (MRC Oulu), Oulu University Hospital and University of Oulu, Oulu, Finland; ^3^Biocenter Oulu, University of Oulu, Oulu, Finland; ^4^Department of Ophthalmology, Helsinki University Hospital, University of Helsinki, Helsinki, Finland; ^5^Research Unit of Biomedicine, Medical Research Center Oulu (MRC Oulu), Oulu University Hospital and University of Oulu, Oulu, Finland

## Abstract

*Aims*. This study investigated the association of autoantibodies binding to oxidized low-density lipoproteins (oxLDL) in diabetic retinopathy (DR).* Methods*. Plasma from 229 types 1 and 2 patients with DR including diabetic macular edema (DME) and proliferative diabetic retinopathy (PDR) was analysed with ELISA-based assay to determine IgA, IgG, and IgM autoantibody levels binding to oxLDL. The controls were 106 diabetic patients without retinopathy (NoDR) and 139 nondiabetic controls (C).* Results*. PDR group had significantly higher IgA autoantibody levels than DME or NoDR: mean 94.9 (SD 54.7) for PDR, 75.5 (41.8) for DME (*p* = 0.001), and 76.1 (48.2) for NoDR (*p* = 0.008). There were no differences in IgG, IgM, or IgA that would be specific for DR or for DME. Type 2 diabetic patients had higher levels of IgA autoantibodies than type 1 diabetic patients (86.0 and 65.5, resp., *p* = 0.004) and the highest levels in IgA were found in type 2 diabetic patients with PDR (119.1, *p* > 0.001).* Conclusions*. IgA autoantibodies were increased in PDR, especially in type 2 diabetes. The high levels of IgA in PDR, and especially in type 2 PDR patients, reflect the inflammatory process and enlighten the role of oxLDL and its autoantibodies in PDR.

## 1. Introduction

Diabetes and its long-term complications continue to represent a severe health problem all around the world. A number of recent studies have emphasized that diabetes carries a strong inflammatory component and the induction of vascular inflammation in diabetes involves a dysregulation of oxidation reaction [1–3]. Elevated plasma levels of circulating oxidized low-density lipoprotein (oxLDL) have been associated with obesity-related metabolic disturbances such as the metabolic syndrome and diabetes [4]. The presence of vascular oxidative stress and low-density lipoprotein (LDL) with increased susceptibility to oxidation is especially prominent in type 2 diabetes [5].

Oxidized low-density lipoproteins are immunogenic [6] and circulating autoantibodies binding to oxidized epitopes of oxLDL have been detected in human and animal plasma [7, 8]. In mouse models of atherosclerosis immunoglobulin M (IgM) type autoantibodies binding to oxLDL have exhibited putative atheroprotective properties [9]. In some studies, the concentrations of serum IgA binding to oxidized LDL have been elevated in subjects with metabolic abnormalities [8] and this phenomenon correlated with plasma levels of inflammatory mediators [10]. Furthermore, plasma IgA autoantibody levels binding to oxLDL have been shown to be positively and IgG autoantibody levels to be negatively associated with markers of glucose metabolism and also to be independent risk factors for type 2 diabetes [8].

The levels of autoantibodies binding to oxLDL decline with age, diabetes duration, and glycated hemoglobin (HbA1c) levels [1, 11]. This phenomenon has been attributed to increased formation of oxLDL-specific immune complexes [12]. It seems that these complexes [13] as well as the oxLDL itself [14] can induce macrophages to be converted into foam cells. The autoantibodies induce the macrophages to produce cytokines [15] which in turn activate endothelial cells and trigger an inflammation cascade [13, 16]. The presence of inflammation and activation of endothelial cells are also key elements in the initiation of diabetic retinopathy (DR) [17, 18]. Previously, oxLDL has been demonstrated to play a role in the development of DR since immunostaining of apolipoprotein B (apoB) oxLDL has been detected in the retinas of type 2 diabetic patients with or without DR and an increase in oxLDL levels reflects the severity of retinopathy [19]. In another study, Fu et al. demonstrated that the oxidative stress was induced by modified LDL in DR; that is, the modified LDL exerted toxic effects on the capillary pericytes [20]. Furthermore, high levels of oxLDL in immune complexes have been shown to associate with progression of retinopathy in type 1 diabetes [21].

Plasma levels of autoantibodies binding to oxLDL might serve as a biomarker for the severity of the diabetic retinopathy, but their role is not yet well characterized. The purpose of this study was to investigate the levels of autoantibodies binding to oxLDL in the plasma of diabetic patients with and without diabetic retinopathy in a homogenous, well-characterized Finnish study population.

## 2. Methods

### 2.1. Study Subjects

**Figure 1:**
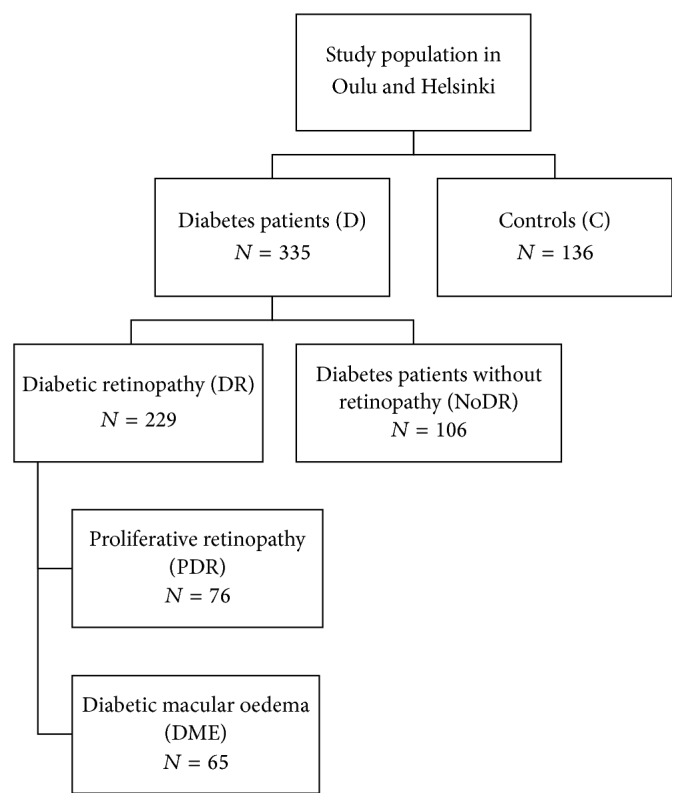
Study population.

This is a case-control study with the study population (Figure 1) consisting of 229 diabetic patients with clinically moderate to severe DR (DR group) from Oulu University Hospital or Helsinki University Hospital and 106 diabetic patients without signs of retinopathy (noDR group) attending fundus imaging for screening of DR in Oulu City Health Centers. The diabetic patients were matched for duration of diabetes by including only those patients with a disease duration of at least 10 years in the noDR group since diabetes' duration and hyperglycemia are the main prognostic factors for the development of DR. The diagnosis and classification of DR were made in the clinical examination and/or from fundus images by experienced ophthalmologist at the Department of Ophthalmology of Oulu and Helsinki University Hospital [22, 23]. We also examined 139 nondiabetic patients undergoing eye surgery in the Helsinki University Hospital as control group (C group).

The DR patients were divided into two groups according to their retinopathy status (Figure 1). Thus the DME group consisted of 65 patients with clinically significant diabetic macular edema (DME) and the proliferative diabetic retinopathy (PDR) group consisted of 76 patients. All subjects from Oulu University Hospital and Oulu City Health Centers filled in a questionnaire and provided blood samples. The questionnaire included questions about their diabetes, diabetic complications, other diseases and medications, and lifestyle. Subjects from the Helsinki University Hospital also provided blood samples and filled in a different type of questionnaire, but therefore some data is missing from the tables and figures. Diabetic patients who had been previously diagnosed with microalbuminuria or proteinuria were classified as having nephropathy and the diagnosis of neuropathy was based on a previous diagnosis according to the questionnaire. This study follows the guiding principles of the Declaration of Helsinki and was approved by the Ethics Committee of the Oulu University Hospital.

### 2.2. Laboratory Measurements

Plasma samples were taken after an overnight fast, centrifuged, and stored −70°C. The levels of fasting plasma glucose, HbA1c, creatinine, total cholesterol, LDL, high-density lipoprotein (HDL), and triglycerides were determined from the blood samples provided by the subjects from Oulu University Hospital and Oulu City Health Centers as routine laboratory measurements in Oulu University Hospital. LDL was extracted from a human plasma sample pool of seven healthy control study subjects of both genders and purified with dialysis [24]. Purified LDL was oxidized for 25 min at +37°C with 0.5 M malondialdehyde (MDA) [6]. The MDA-solution used was produced from the MDA-base solution incubated with 4 N hydrochloric acid in +37°C for 12–15 minutes until turning yellow and neutralized with 1 N sodium hydroxide.

### 2.3. Autoantibody Measurements

Plasma IgG, IgM, and IgA class autoantibody titers to oxidized LDL were measured with chemiluminescence ELISA as previously described [25]. The measurement plates were coated with MDA-oxLDL and incubated overnight at +4°C. The wells were washed with a buffer solution (phosphate buffered saline with ethylenediaminetetraacetic acid (PBS-EDTA)) and postcoated with 0.5% FISH-gelatin for one hour at room temperature. After a second wash with PBS-EDTA, samples in 0.5% FISH-gelatin were added and incubated overnight at +4°C. Each plate contained also a triplicate standards of commercial purified immunoglobulins as controls (Sigma Aldrich, St. Louis, MO), a zero-sample of pure PBS-EDTA-FISH-gelatin, and two triplicate control samples (“high” and “low”), diluted to cover as wide a range of the standard as possible. After a third wash, the anti-human antibodies in 0.5% FISH-gelatin were added and incubated for one hour at room temperature. Subsequently the plates were washed with PBS-EDTA and distilled water and then they were incubated with 0.33% LUMIPHOS (Lumigen Inc. Southfield, MI) for 90 minutes and analysed in a VICTOR multilabel counter (PerkinElmer, Waltham, MA).

In order to maintain the sample luminescence counts within the standard range, the samples were diluted in FISH-gelatin. Prior to the final analysis, we performed multiple tests to determine the dilutions for each autoantibody type. The selection of the dilutions was based on the lowest coefficient of variation (CV) between measurements. The dilutions were 1 : 2000 for IgG, 1 : 1333 for IgM, and 1 : 400 (MDA-ox) or 1 : 100 (Cu-ox) for IgA. We performed triplicate measurements of each sample. The CV was calculated from each triplicate measurement. The CVs were below 20% in all samples.

An average relative light unit (RLU) value was calculated from the luminescence counts by reducing the blank value (zero-sample) from the average luminescence count of the triplicate measurements. A linear standard curve was created and the RLUs were converted into relative plasma autoantibody levels by dividing the RLUs with the standard curve slope and then multiplied by the dilution coefficient. The levels are expressed as relative units (RU).

### 2.4. Statistical Analyses

Statistical analysis was performed with the IBM SPSS software (IBM Corporation, Armonk, NY). The statistical significance of the autoantibody levels between two study groups was calculated with independent samples *t*-test and ANOVA was used for comparisons between several study groups. Crosstabs (Chi-square) was used to assess differences between categorical variables. Multiple linear regression analysis was used to explain the levels of autoantibodies and variables included in the model were selected due to correlation (Pearson correlation) with autoantibody levels (sex, age, BMI, diabetes duration and type, gHbA1c, LDL, and medications). *p* values less than 0.05 were considered statistically significant.

## 3. Results

### 3.1. Baseline Characteristics

The clinical characteristics of the study groups are shown in Tables 1 and 2. The DR (*n* = 229) and NoDR (*n* = 106) groups did not differ significantly in terms of age, sex, body mass index (BMI), diabetes type or LDL, and total cholesterol concentrations, but the DR group had worse diabetes control (Table 1) than the NoDR group, with the mean value of glycated hemoglobin being higher in the DR group. The DR group had poorer lipid profile having higher triglyceride and lower HDL concentration than the NoDR group (Table 1).

The mean age of the patients in the DME group was older than in the PDR group (63.6 and 55.4 years, resp., *p* < 0.001) (Table 2). As expected, the proportion of patients with type 2 diabetes was higher in the DME group than in the PDR group (72.3% and 39.5% of patients in DME and PDR, respectively (*p* < 0.001)) but there was some overlapping. There were no differences in other measured clinical characteristics between the groups (Table 2), except that more patients suffered from nephropathy (microalbuminuria) in the PDR group as compared to the DME group (42.9% versus 23.8%, *p* = 0.020). The medications the diabetic subjects used are shown in Table 3. The diabetic patients, according to clinical guidelines, had medications influencing blood pressure and lipid profile in addition to antidiabetic drugs and the percentage of patients having beta blocker, ACE inhibitor, and statin medications was higher in DR group than in NoDR group. No differences in insulin, oral diabetes medication, or ASA were found between DR and NoDR.

### 3.2. Autoantibody Levels in DR

**Figure 2:**
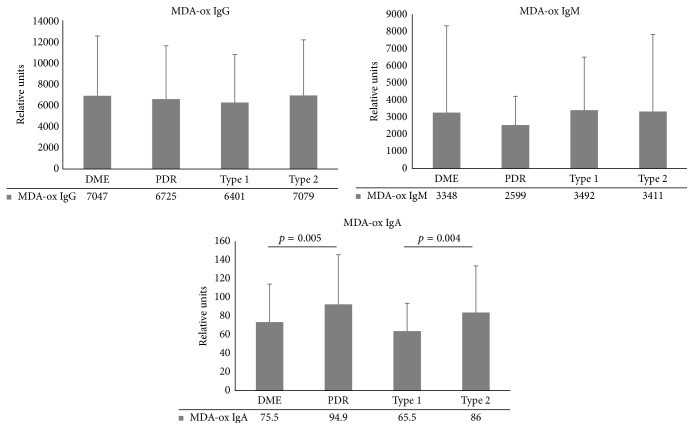
Autoantibody levels against MDA-oxLDL (MDA-Ox IgG, MDA-Ox IgM, and MDA-Ox IgA) in macular edema patients (DME), proliferative retinopathy (PDR), and type 1 and type 2 diabetes patients. The levels are expressed as mean relative units and standard deviation.

Retinopathy did not influence the measured autoantibody levels: IgG, IgM, or IgA; autoantibody levels did not differ significantly between the DR and noDR groups (*p* = 0.644, *p* = 0.579, and *p* = 0.346, resp.) (Table 1, Figure 2). However, PDR group had significantly increased IgA autoantibody levels; that is, the mean value of IgA was 94.9 (SD 54.7) compared with 75.5 (SD 41.8) in DME (*p* = 0.023) (Figure 2) and 76.1 (SD 48.2, *p* = 0.008) in NoDR (Table 1).

### 3.3. Autoantibody Levels in Diabetes

We also wanted to assess the effect of diabetes on autoantibody levels. Diabetes influenced IgM autoantibody levels: diabetic patients (both DR and NoDR) had significantly lower IgM autoantibody levels against MDA-oxLDL than nondiabetic controls (3389 (SD 3998) versus 4258 (SD 3578), *p* = 0.043), but the IgG and IgA autoantibody levels did not differ significantly between the D group (DR and NoDR) and the C group. The levels for for IgM, IgG, and IgA were 3389 (SD 3998), 6944 (SD 5280), and 79.6 (SD 46.3) for D group and 4258 (SD 3578), 6874 (SD 4718), and 80.7 (SD 46.2) for C group, respectively.

### 3.4. Effect of Diabetes Type on Autoantibody Levels

**Figure 3:**
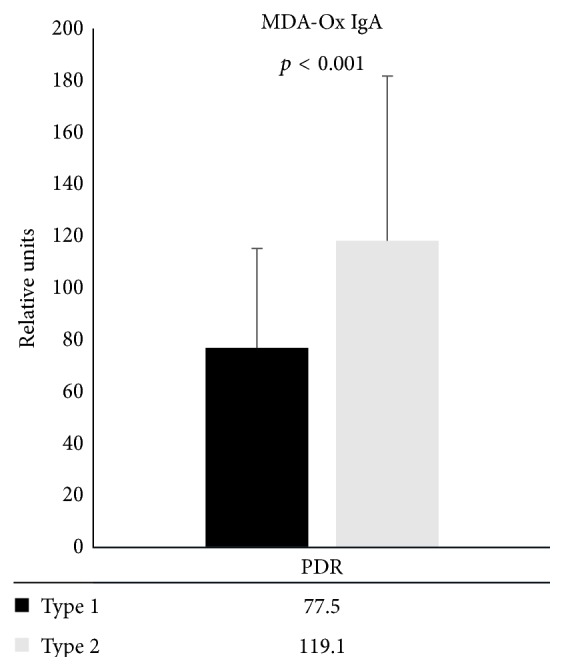
IgA autoantibody levels against MDA-oxLDL (MDA-Ox IgA) in macular edema patients (DME) and proliferative retinopathy (PDR) patients divided by diabetes types (type 1 and type 2). The levels are expressed as mean relative units and standard deviation.

The mean age of type 1 diabetic patients was 45.7 years (SD 13.5) and of type 2 diabetic patients was 66.8 (SD 9.6). We subdivided them according to type of diabetes, and it was found that the IgA autoantibody levels were significantly lower in type 1 diabetes than in type 2 diabetes (65.5 (SD 30.5) for type 1 and 86.0 (SD 51.3) for type 2, *p* < 0.001) (Figure 2). We further tested the effect of diabetes type in PDR group and found that the IgA levels were highest in the PDR group having type 2 diabetes (119.1 (SD 64.1) versus 77.5 (SD 38.7) in PDR type 1 population (*p* = 0.002)) (Figure 3).

### 3.5. Multiple Linear Regression

Multiple linear regression was run to test the main determinants of autoantibody levels. Variables in the model were sex, age, BMI, diabetes duration and type, gHbA1c, LDL, and medications. The variables that added statistically significantly to the equation are shown in Table 4. In general, IgG autoantibodies were increased by type 2 diabetes and decreased by oral diabetes medication and statin medication (*R*^2^ = 0.122). High LDL concentration influenced IgM levels and they were decreased by female sex and oral diabetes medication (*R*^2^ = 0.161). Furthermore, it was found that IgA autoantibody levels were increased by increasing age, gHbA1c, LDL, and ASA medication (*R*^2^ = 0.227).

## 4. Discussion

In this study, we have investigated the autoantibody levels against MDA-OxLDL in diabetic retinopathy, an observational analysis using a cross-sectional study design, and found that the levels of IgA type autoantibodies were increased in PDR patients as compared with DME or noDR and the type 2 patients in PDR group had the highest levels.

There is evidence that autoantibodies binding to oxLDL are involved in the pathogenesis of DR and it may be that its tissue-specific function is related to immune complexes. It has been shown by Wu et al. that oxidized LDL measured as intraretinal immunofluorescence of apoB-100 (the protein component of LDL) is present in human donor diabetic retinas and the level of immunofluorescence increases with the severity of DR [19]. Even at the earliest stages of DR before the onset of clinical retinopathy, an aggregation of oxidized LDL has been detected in the retina. The oxidized LDL is also an effective trigger of apoptosis in retinal capillary pericytes leading to the breakdown of the blood-retina barrier, a key event in the development of DR [26]. Extravasated modified lipoproteins may play a key role in the development of DR; the phenomenon is described in human, animal, and cell culture studies [20] indicating that lipoproteins may be potential causal factors in the development of DR and that the pathogenic process occurring in DR might be somewhat parallel to that observed in atherosclerosis. Furthermore, OxLDL may either be important in the pathogenesis of DR per se or act as a trigger for inflammation in the retina and in the surrounding retinal vessels. It has been shown that modified lipoproteins activate innate and adaptive immune responses with proinflammatory signals and disturb the integrity of the microvasculature [27]. Also the study of Fu et al. supports this hypothesis, since oxLDL-immune complexes triggered apoptosis and enhanced inflammatory cytokine secretion towards retinal pericytes [28]. Furthermore, it seems that oxLDL and autoantibodies might also be important in prognosis of DR as oxLDL-immune complexes and advanced glycation end products modified low-density lipoproteins (AGE-LDL) were associated with an increased risk of progression to advanced retinopathy in patients with type 1 diabetes [21].

As stated, there seem to be several similarities in the role of oxLDL in the pathogenesis of DR and atherosclerosis. There is evidence in humans that the IgM antibodies binding to oxLDL might have an atheroprotective effect as shown in human and mouse studies [9, 24], although with controversy [29]. It seems that instead of being an independent risk factor in atherosclerosis, IgM autoantibodies may modulate the risk of coronary artery disease associated with elevated levels of oxidative biomarkers [30]. The role of IgA autoantibodies binding to oxLDL in atherosclerosis still remains somewhat unclear. It has been postulated that high autoantibody levels binding to oxLDL could well be useful clinical parameters of lipoprotein oxidation for detecting the presence of macrovascular disease in diabetic patients [31]. Our results suggest that determining plasma autoantibody levels against oxLDL might represent a potential indicator for diabetic retinopathy, but this deserves further studies.

Plasma levels of autoantibodies against oxLDL have also been shown to correlate with diabetes or diabetes risk. Previously, low levels of oxLDL antibodies, especially of the IgG type autoantibodies, have been associated with type 2 diabetes, since a low total oxLDL autoantibody level has been linked with the development of type 2 diabetes in women [32] and inversely correlating with markers of glucose metabolism [8]. Furthermore, a harmful role has been proposed for IgA autoantibodies, since higher levels of IgA autoantibodies increased the risk of diabetes in a population-based cohort although no association was found between the levels of IgM autoantibodies and glucose metabolism in a previous study [8]. We found that the IgA levels were highest in type 2 diabetes among PDR group, indicating that the higher levels found were not only disease-specific but also diabetes-type-specific. Previously, it has been shown that these IgA autoantibodies were associated with inflammatory markers, obesity, and type 2 diabetes [10]. In PDR the inflammation and oxidative processes are more active than in DME and it seems that, in type 2 diabetes with PDR, these processes might be further increased as a consequence to local (or general) oxidative stress. It seems that IgA autoantibodies to oxLDL might have a role in the complications encountered in type 2 diabetes, at least at the microvascular level. Other types of autoantibodies seem to be associated with other types of diabetes complications, since IgG type autoantibody was increased in type 1 diabetes [33] and may have a pathogenic role in the development of nephropathy [34].

In our case-control study, we observed a significant increase in levels of IgA autoantibodies in PDR as compared to NoDR or DME groups and this increase was most prominent in type 2 PDR patients. This is the first analysis of autoantibody levels binding to oxLDL in patients with type 2 diabetes and retinopathy. In the present study, a relatively large number of well-characterized patients including both type 1 and type 2 diabetic patients with both DME and PDR were studied. Nonetheless, since diabetes control, retinopathy screening and treatment of DR are stringently regulated in Finland, the study population is rather homogenic. Some limitation of the current study might be that the levels of free autoantibodies and the autoantibodies bound to oxLDL forming immunocomplexes [10] may vary between individuals. Our method will not measure total autoantibody levels to or reflect the total immune response to the antigen. This is a universal phenomenon in measuring the circulating antibodies in plasma samples. In addition, the plasma levels of oxLDL autoantibodies are not specific for retinal damage might also be influenced by the severity of atherosclerosis and/or renal disease or might be affected by influence of diabetes on the vascular wall [35]. There is also some contrasting evidence about using anti-oxLDL as a marker, as antibody concentrations might reflect individual immunity strength, which remains to be solved in future studies.

## 5. Conclusions

Our study shows that the plasma levels of IgA type autoantibodies were increased in PDR and especially the type 2 patients in PDR group had the highest levels. Our results highlight the role of oxLDL and its autoantibodies in PDR and suggest that they might have relevance as an indicator of DR. Our understanding of oxLDL in the pathogenesis of diabetic retinopathy is increasing but there are still unexplored areas. Clarifying the role of inflammation and immunity in the development of diabetic vascular complications deserves increased attention and may bring tools for DR prevention and treatment.

## Figures and Tables

**Table 1:** Clinical characteristics and levels of MDA-ox LDL of diabetic patients with diabetic retinopathy (DR) and without diabetic retinopathy (noDR). The data are expressed as mean (standard deviation (SD)) or *n* (percent (%)).

	DR	NoDR	*p*
	*n* = 229	*n* = 106	
Age (years)	58.9 (14.4)	55.9 (16.3)	0.126
Gender			0.411
Women	97 (42.4%)	50 (47.2%)	
Men	132 (57.6%)	56 (52.8%)	
BMI (kg/m^2^)	28.8 (5.8)	27.2 (5.0)	0.056
Diabetes			0.636
Type 1	98 (43.8%)	50 (47.2%)	
Type 2	126 (56.2%)	56 (52.8%)	
Duration	22 (11.5)	24.2 (7.8)	0.046
BP systolic (mmHg)	152.2 (23.6)	133.9 (13.5)	0.032
BP diastolic (mmHg)	83.8 (11.8)	83.6 (10.1)	0.976
Nephropathy	64 (37.2%)	16 (15.8%)	<0.001
Neuropathy	68 (40.5%)	19 (18.4%)	<0.001
Hypertension	171 (76.7%)	55 (51.9%)	<0.001
Cholesterol (mMol)	4.1 (1.1)	4.1 (0.9)	0.553
LDL (mMol)	2.3 (0.9)	2.1 (0.8)	0.122
HDL (mMol)	0.9 (0.5)	1.1 (0.5)	<0.001
Triglycerides (mMol)	1.2 (1.4)	0.6 (0.8)	<0.001
Creatinine (*μ*Mol)	102 (82.8)	72.9 (21.8)	<0.001
Glucose (mMol)	8.9 (4.0)	8.0 (2.9)	0.029
HbA1c (%)	8.5 (1.8)	7.7 (1.3)	<0.001
MDA-ox IgG	6827 (5397)	7177 (5056)	0.579
MDA-ox IgM	3316 (4489)	3536 (2933)	0.644
MDA-ox IgA	81.3 (45.4)	76.1 (48.2)	0.346

BMI: body mass index, BP: blood pressure, HbA1c: glycated hemoglobin, HDL: high-density lipoprotein, and LDL: low-density lipoprotein.

**Table 2:** Clinical characteristics of the diabetic retinopathy patients (DR) with diabetic macular edema (DME) or proliferative retinopathy (PDR). The data are expressed as mean (standard deviation (SD)) or *n* (percent (%)).

	DME	PDR	*p*
	*n* = 65	*n* = 76	
Age (years)	63.6 (10.2)	55.4 (15.1)	<0.001
Gender			0.272
Women	29 (44.6%)	27 (35.5%)	
Men	36 (55.4%)	49 (64.5%)	
BMI (kg/m^2^)	30.1 (5.8)	28.2 (5.6)	0.057
Diabetes			<0.001
Type 1	18 (27.7%)	46 (60.59%)	
Type 2	47 (72.3%)	30 (39.5%)	
Duration	22.1 (9.5)	24.9 (10.2)	0.095
BP systolic (mmHg)	145.4 (24.1)	152.2 (20.6)	0.448
BP diastolic (mmHg)	78.3 (7.1)	83.9 (13.9)	0.305
Nephropathy	15 (23.8%)	30 (42.9%)	0.020
Neuropathy	21 (33.9%)	32 (46.4%)	0.145
Hypertension	54 (84.4%)	55 (78.6%)	0.389
Cholesterol (mMol)	4.6 (1.1)	4.7 (1.1)	0.496
LDL (mMol)	2.7 (0.9)	2.8 (0.9)	0.737
HDL (mMol)	1.3 (0.3)	1.3 (0.4)	0.966
Triglycerides (mMol)	1.5 (1.0)	2.0 (1.7)	0.086
Creatinine (*μ*Mol)	91.6 (64.2)	114.0 (103.7)	0.124
Glucose (mMol)	8.9 (3.9)	9.3 (3.9)	0.521
HbA1c (%)	9.2 (2.1)	9.1 (1.7)	0.657

BMI: body mass index, BP: blood pressure, HbA1c: glycated hemoglobin, HDL: high-density lipoprotein, and LDL: low-density lipoprotein.

**Table 3:** Percentages of diabetic patients using lipid lowering, antihypertensive, oral diabetes medication, insulin, or ASA.

	Yes		No		Missing	
	*N*	%	*N*	%	*N*	%
ACE/ATII	164	49.0	151	45.1	20	6.0
*β*-Blocker	113	33.7	210	62.7	12	3.6
ASA	104	31.0	219	65.4	12	3.6
Statin	109	32.5	210	62.7	16	4.8
Oral DM medication	121	36.1	205	61.2	9	2.7
Insulin	215	64.2	55	16.4	65	19.4

**Table 4:** Multiple linear regression for autoantibody levels. The variables included in the model were sex, age, BMI, diabetes duration and type, gHbA1c, LDL, and medications. Negative values indicate inverse effect and for sex, female sex has decreasing effect.

Variable	*B*	95% confidence interval	*p*
MDAOx-IgG			
Constant	5070.0		**0.005**
Oral diabetes medication	−3857.0	−5693.9 to −2020.1	<0.001
Diabetes type	3624.4	1590.1 to 5658.0	0.001
Statin medication	−1954.9	−3495.4 to −414.3	0.013
MDAOx-IgM			
Constant	−1777.7		**<0.001**
Sex	−995.8	−1912.8 to −78.69	0.033
LDL	919.5	314.5 to 1524.4	0.003
Oral diabetes medication	−2038.5	−3415.0 to −662.0	0.004
MDAOx-IgA			
Constant	−40.93		**<0.001**
Age	0.84	0.37 to 1.32	0.001
gHbA1c	3.64	0.44 to 6.87	0.026
ASA	21.91	8.20 to 35.62	0.003
LDL	8.08	0.91 to 15.25	0.027
